# mRNA and microRNA Expression Profile of Corneal and Conjunctival Impression Cytology Samples

**DOI:** 10.3390/biology15151239

**Published:** 2026-07-27

**Authors:** Shuailin Li, Tanja Stachon, Fabian Norbert Fries, Berthold Seitz, Nicole Ludwig, Nóra Szentmáry

**Affiliations:** 1 Dr. Rolf M. Schwiete Center for Limbal Stem Cell and Congenital Aniridia Research, Saarland University, 66424 Homburg, Germany; shuailin.li@uni-saarland.de (S.L.); tanja.stachon@uni-saarland.de (T.S.); fabian.fries@uks.eu (F.N.F.); 2 Department of Experimental Ophthalmology, Saarland University, 66424 Homburg, Germany; 3 Department of Ophthalmology, Saarland University Medical Center, 66424 Homburg, Germany; berthold.seitz@uks.eu; 4 Department of Human Genetics, Saarland University, 66424 Homburg, Germany; nicole.ludwig@uni-saarland.de; 5 Department of Ophthalmology, Semmelweis University, 1085 Budapest, Hungary

**Keywords:** cornea, conjunctiva, impression cytology, mRNA, microRNA, bioinformatics analysis

## Abstract

**Purpose:** To characterize the messenger RNA (mRNA) and microRNA (miRNA) expression profiles of the normal human cornea and conjunctiva using impression cytology (IC) samples and to investigate their molecular characteristics and regulatory networks. **Methods:** Corneal and conjunctival IC samples were collected from healthy subjects. Whole-transcriptome and miRNA sequencing were performed, followed by differential expression and bioinformatics analyses. Regulatory networks, protein interaction networks, and functional enrichment analyses were constructed. Selected genes and miRNAs were validated by RT-qPCR. **Results:** A total of 1676 differentially expressed genes and 175 differentially expressed miRNAs were identified between the cornea and conjunctiva. Functional analyses revealed that genes showing higher expression in the cornea were mainly associated with epithelial structure, barrier function, and antiviral immune responses. In contrast, genes showing higher expression in the conjunctiva were primarily involved in immune regulation, secretion, metabolic detoxification, and tissue remodeling. PPI network analysis showed that hub genes in the cornea were predominantly interferon-stimulated genes related to antiviral responses, while those in the conjunctiva were mainly involved in cell cycle regulation and metabolic detoxification. GO and KEGG analyses further supported these functional distinctions. RT-qPCR validation generally supported the expression patterns identified by RNA sequencing. **Conclusions:** Our findings reveal that the cornea is characterized by gene expression programs supporting epithelial homeostasis, barrier function, and antiviral immunity, whereas the conjunctiva exhibits transcriptional signatures related to immune surveillance, secretion, metabolic processing, and tissue remodeling. The miRNA–mRNA regulatory networks constructed in this study provide new insights into the molecular regulatory mechanisms of the ocular surface and offer a theoretical basis for future research into disease mechanisms and targeted therapeutic strategies.

## Simple Summary

The cornea and conjunctiva form the ocular surface and are essential for maintaining vision and protecting the eye from environmental challenges. Although these tissues work closely together, they serve distinct functions, and the molecular mechanisms underlying their specialization are not fully understood. In this study, we analyzed mRNA and microRNA expression profiles in healthy human corneal and conjunctival samples collected by impression cytology. We found that the mRNA and microRNA expression profiles of the cornea were enriched for pathways related to epithelial integrity, barrier function, and antiviral defense, whereas those of the conjunctiva were enriched for pathways associated with immune surveillance, secretion, detoxification of environmental substances, and tissue repair. We also identified microRNA–mRNA regulatory networks that may contribute to these tissue-specific functions. Together, these findings provide a comprehensive molecular reference for the healthy ocular surface and improve our understanding of the biological differences between the cornea and conjunctiva. This knowledge may support future research into ocular surface diseases and facilitate the discovery of novel biomarkers and therapeutic targets.

## 1. Introduction

The cornea and conjunctiva, which form the outermost structures of the anterior ocular surface, are essential components of the ocular surface system. Together, they maintain the stability of the ocular surface microenvironment and support normal visual function [1,2]. The cornea represents a critical element of the eye’s refractive apparatus and serves as the primary interface for incoming light. The corneal epithelium, the outermost cellular layer of the cornea, possesses substantial regenerative capacity and can rapidly repair itself following injury. In addition to providing an important protective barrier for the underlying ocular tissues, its interaction with the tear film plays a pivotal role in preserving the structural and functional integrity of the ocular surface [3,4].

The conjunctiva is a translucent mucous membrane covering part of the anterior surface of the eyeball and the inner surface of the eyelids. It plays essential roles in immune defense, lubrication, and maintenance of tear film stability [5,6]. Various ocular surface diseases, including dry eye disease, keratoconjunctivitis, and autoimmune-associated ocular disorders, may lead to structural and functional alterations in both the cornea and conjunctiva. The development of these conditions is frequently accompanied by complex molecular regulatory mechanisms [7,8,9].

In recent years, increasing attention has been directed toward the roles of messenger RNA (mRNA) and microRNA (miRNA) in ocular surface diseases. As the direct template for protein synthesis, mRNA conveys genetic information from DNA to proteins, and its expression levels can directly influence cellular function and physiological state [10]. In contrast, miRNAs are small non-coding RNA molecules, typically 20–24 nucleotides in length, that regulate target gene expression at the post-transcriptional level and are involved in processes such as cell differentiation, proliferation, apoptosis, and inflammatory responses [11,12]. Accumulating evidence indicates that miRNAs play critical roles in the regulation of ocular surface inflammation, cell survival, and epithelial repair, and that dysregulated miRNA expression is closely associated with various ocular surface diseases [13,14,15]. However, systematic investigations of mRNA and miRNA expression profiles in both the corneal and conjunctival surface remain limited, particularly studies based on integrated analyses of clinical samples.

Ocular surface impression cytology (IC) is a minimally invasive and repeatable technique that enables rapid collection of superficial cells from the cornea and conjunctiva and has been widely used in the diagnosis and investigation of ocular surface diseases [16]. The method is simple to perform, well tolerated by patients, and provides cellular samples that reliably reflect ocular surface alterations, thereby serving as suitable material for subsequent RNA- and protein-based analyses [17].

With advances in high-throughput sequencing and gene expression profiling technologies, the use of IC samples for integrated analyses of mRNA and miRNA expression profiles has emerged as an important approach for investigating the molecular mechanisms underlying ocular surface diseases [18,19]. In the present study, corneal and conjunctival IC samples were obtained from healthy subjects using the EYEPRIM™ device (OPIA Technologies, Paris, France) [20], followed by a systematic analysis of mRNA and miRNA expression profiles to explore their potential regulatory networks in ocular surface physiology and pathology. By integrating the expression characteristics of these two RNA classes, this study may contribute to a better understanding of the coordinated and differential molecular regulatory mechanisms between the cornea and conjunctiva and may provide novel insights into early diagnosis, biomarker identification, and targeted therapeutic strategies for ocular surface diseases.

## 2. Materials and Methods

### 2.1. Ethical Considerations

The present study was carried out in compliance with the Declaration of Helsinki. Informed consent in written form was obtained from all participants or their legal guardians prior to inclusion. Ethical approval was granted by the Ethics Committee of Saarland, Germany (No. 110/17).

### 2.2. Corneal and Conjunctival IC Sample Collection

Corneal and conjunctival IC samples were obtained from healthy donors at the Department of Ophthalmology, Saarland University Medical Center, Homburg/Saar, Germany. Corneal and conjunctival samples were obtained from different individuals. Before enrollment, all healthy participants underwent a comprehensive ophthalmic examination and medical history assessment. Individuals with ocular diseases, systemic conditions that could affect the ocular surface, a history of ocular surgery, topical ocular medication use, or contact lens wear were excluded. These eligibility criteria were applied to minimize potential confounding factors that could influence ocular surface gene expression. Sample collection was carried out in accordance with the manufacturer’s instructions for the EYEPRIM™ device (Opia Technologies, Paris, France).

Briefly, topical anesthesia was induced by instillation of 0.4% oxybuprocaine hydrochloride eye drops into the conjunctival sac. IC samples were subsequently collected using the EYEPRIM™ device, which provides a standardized sampling area of approximately 69 mm^2^. The applicator tip was gently applied to the corneal or conjunctival surface, and light pressure was maintained for 2–3 s. The device was then vertically removed to retrieve the cellular specimen. Immediately after collection, the membrane was transferred into lysis buffer provided with the DNA/RNA/Protein Purification Kit Micro (Norgen Biotek Corp., Thorold, ON, Canada) and stored at −80 °C until RNA extraction.

In total, corneal IC samples were collected from 14 healthy subjects (mean age: 29.0 ± 19.4 years; range: 2–60 years; 7 males and 7 females), whereas conjunctival IC samples were obtained from 10 healthy subjects (mean age: 31.5 ± 11.1 years; range: 22–60 years; 5 males and 5 females). No significant differences were observed between the two groups with respect to age (Mann–Whitney test, *p* = 0.3944) or sex distribution (Chi-square test, *p* > 0.9999). Detailed demographic characteristics of the study population are summarized in Table 1.

### 2.3. RNA Isolation and Assessment of RNA Concentration and Purity

Total RNA, including miRNAs, was isolated from all corneal and conjunctival IC samples using the DNA/RNA/Protein Purification Kit Micro (Norgen Biotek Corp., Thorold, ON, Canada) according to the manufacturer’s instructions. The purified RNA was eluted in 30 μL of nuclease-free water and stored on ice for immediate analysis or at −80 °C for subsequent experiments. RNA concentration and purity were assessed using a UV/VIS spectrophotometer (Analytik Jena AG, Jena, Germany). All corneal and conjunctival samples met the quality requirements for RNA sequencing, with sufficient RNA concentration and purity for downstream transcriptomic analyses.

### 2.4. Whole-Transcriptome and miRNome Sequencing and Data Analysis

Global transcriptome profiling was performed using RNA isolated from 14 corneal and 10 conjunctival IC samples. The specific procedure was described in detail in our previous study [21]. All samples were processed using the same standardized protocols for RNA extraction, library preparation, sequencing, and downstream bioinformatic analyses under identical experimental conditions to minimize technical variability. RNA sequencing libraries were prepared using the MGIEasy rRNA Depletion Kit in combination with the MGIEasy Universal Library Preparation Set (MGI Tech, Shenzhen, China) according to the manufacturer’s instructions. Sequencing was carried out at the Sequencing Unit of the Core Facility for Molecular Single Cell and Particle Analysis, Saarland University, using the DNBSEQ-G400RS platform to generate 100 bp paired-end reads.

Bioinformatic processing and quantification of RNA-Seq data were performed using the mRNA workflow implemented in snakePipes (version 2.8.1) [22]. Sequencing reads were aligned to the human reference genome (GRCh38) using STAR (version 2.7.11b) [23], and gene-level quantification was conducted with FeatureCounts (version 2.0.6) [24]. Sequencing quality was assessed using FastQC (version 0.12.1), and summary quality reports were generated with MultiQC (version 1.27.1) to evaluate overall dataset quality [25]. For downstream analyses, raw count data were normalized using variance-stabilizing transformation (VST) implemented in DESeq2 (version 1.46.0) [26], and the transformed data were used for clustering analyses. Differential gene expression analysis was performed in DESeq2 using raw count data, and log2 fold changes (FCs) were calculated for each gene. Statistical significance was determined following multiple testing correction using the Benjamini–Hochberg method, with a false discovery rate (FDR) threshold of 0.05.

For miRNA profiling, sequencing libraries were prepared using the MGIEasy Small RNA Library Prep Kit (MGI Tech, Shenzhen, China) according to the manufacturer’s protocol. Sequencing was performed on the DNBSEQ-G400RS platform, generating 50 bp single-end reads. Raw sequencing data (FASTQ files) were analyzed using the miRMaster 2.0 pipeline [27], which includes adapter trimming, read collapsing, and miRNA quantification based on the miRBase v22.1 database [28]. To enable comparisons across samples, miRNA expression levels were normalized as reads per million mapped miRNAs (RPMMM).

Principal component analysis (PCA) was performed to assess the overall transcriptomic relationships among corneal and conjunctival impression cytology (IC) samples. PCA was conducted using the variance-stabilized normalized expression matrix of mRNAs and miRNAs with the FactoMineR package in R (version 4.2.1). The first two principal components (PC1 and PC2) were visualized using the ggplot2 package (version 4.0.3).

### 2.5. Prediction of miRNA–mRNA Regulatory Relationships

Target genes of differentially expressed miRNAs were identified using the online platform miRTarLink 2.0 (https://ccb-compute.cs.uni-saarland.de/mirtargetlink2; accessed on 20 April 2026) [29]. Only miRNA–target interactions supported by strong experimental evidence were included in the analysis, including interactions validated by luciferase reporter assays, immunohistochemistry, microarray analysis, real-time quantitative PCR (RT-qPCR), and Western blotting.

### 2.6. Protein–Protein Interaction (PPI) Network Construction and Hub Gene Identification

To investigate potential functional interactions, PPI networks were constructed for differentially expressed mRNAs and for the predicted target genes of differentially expressed miRNAs using the STRING database (https://string-db.org; accessed on 20 April 2026). An interaction confidence score threshold of 0.7 was applied. PPI networks were visualized using Cytoscape software (version 3.10.4). Key regulatory genes within the networks were identified using the cytoHubba plugin in Cytoscape by applying maximal clique centrality (MCC) analysis. The top 10 hub genes were selected based on their MCC scores.

### 2.7. Gene Ontology (GO) and Pathway Enrichment Analysis

To investigate the potential biological functions of differentially expressed genes (DEGs) and the target genes of dysregulated miRNAs, functional enrichment analyses were performed. Gene Ontology (GO) analysis, including biological process (BP), molecular function (MF), and cellular component (CC) categories, as well as Kyoto Encyclopedia of Genes and Genomes (KEGG) pathway analysis, were conducted using the DAVID online bioinformatics platform (https://davidbioinformatics.nih.gov; accessed on 20 April 2026).

### 2.8. RT-qPCR Validation

RT-qPCR validation was performed using a subset of the RNA samples included in the transcriptomic analyses. Specifically, eight corneal samples were selected from the 14 corneal samples, and eight conjunctival samples were selected from the 10 conjunctival samples used for RNA sequencing based on RNA availability and quality.

For mRNA validation, 500 ng of total RNA was reverse-transcribed into complementary DNA (cDNA) using the OneTaq RT-PCR Kit (New England Biolabs GmbH, Frankfurt am Main, Germany). Quantitative PCR (qPCR) was subsequently performed on a QuantStudio 5 Real-Time PCR System (Applied Biosystems, Waltham, MA, USA) using ACEq SYBR Green Master Mix (Vazyme Biotech, Nanjing, China). Glucuronidase beta (GUSB) and TATA-box binding protein (TBP) were used as endogenous reference genes for normalization.

For miRNA validation, reverse transcription was carried out using the miRCURY LNA miRNA RT Kit, followed by duplicate qPCR analyses using the miRCURY LNA miRNA SYBR Green PCR Kit (Qiagen GmbH, Hilden, Germany) according to the manufacturer’s instructions. miR-103a-3p and small nuclear RNA U6 (RNU6) served as endogenous controls for normalization.

Relative expression levels were calculated using the 2^−ΔΔCT^ method, with conjunctival samples used as the reference group (fold change = 1). Data distribution was assessed using the Shapiro–Wilk test. As the data were not normally distributed, differences in expression levels between corneal and conjunctival samples were analyzed using the Mann–Whitney test.

## 3. Results

### 3.1. mRNA and miRNA Expression Profile

#### 3.1.1. mRNA Sequencing of Corneal and Conjunctival IC Samples

**Figure 1:**
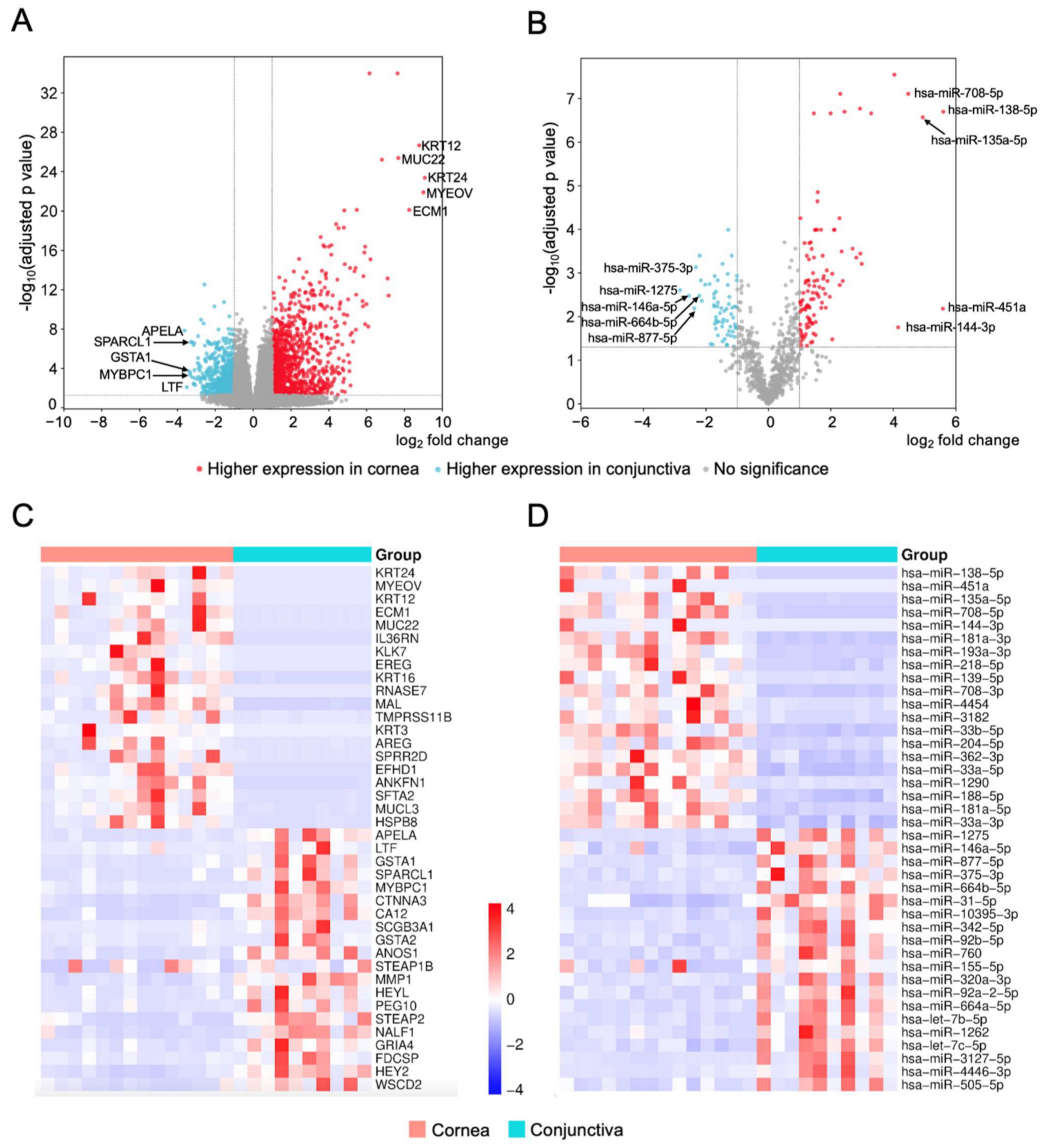
Volcano plots and heatmaps for the differentially expressed mRNAs (**A**,**C**) and miRNAs (**B**,**D**) in corneal impression cytology (IC) samples versus conjunctival IC samples from healthy subjects. The volcano plots display the −log10 (adjusted *p* values) from the *t*-test on the Y-axis and the log2 (fold change) on the X-axis. mRNAs or miRNAs with a log2 (fold change) > 1 and a adjusted *p* value < 0.05 were considered higher expression in cornea, and with a log2 (fold change) < −1 and an adjusted *p* value < 0.05 were considered higher expression in conjunctiva (**A**,**B**). Heatmap rows are labeled with Entrez gene symbols or official miRNA names. Expression intensities are represented by a color gradient ranging from red (maximum expression) through white (average expression) to blue (minimum expression) (**C**,**D**).

mRNA sequencing was performed on normal corneal and conjunctival IC samples to compare transcriptomic expression profiles between the two ocular surface tissues. DEGs were identified using the thresholds of fold change (FC) < −2.0 or >2.0 and an adjusted *p* value < 0.05. Among the 16,091 genes analyzed, a total of 1676 DEGs were identified. Of these, 1082 genes showed higher expression in corneal IC samples than in conjunctival IC samples, whereas 594 genes were more highly expressed in conjunctival samples than in corneal samples (Figure 1A).

Figure 1C and Table 2 summarize the top 20 genes with higher expression in corneal IC samples and the top 20 genes with higher expression in conjunctival IC samples. Among the identified DEGs, *KRT24* showed the highest expression in corneal IC samples and exhibited the largest expression difference between the two tissue samples, with a log2 FC of 9.08. In contrast, *APELA* showed the highest expression in conjunctival IC samples, with a log2 FC of −3.63.

#### 3.1.2. miRNA Sequencing of Corneal and Conjunctival IC Samples

Similarly, miRNA sequencing analysis identified 175 differentially expressed miRNAs among the 982 miRNAs analyzed. Of these, 110 miRNAs showed higher expression in corneal IC samples than in conjunctival IC samples, whereas 65 miRNAs showed higher expression in conjunctival samples than in corneal samples (Figure 1B).

Figure 1D and Table 3 present the top 20 miRNAs with higher expression in corneal IC samples and the top 20 miRNAs with higher expression in conjunctival IC samples. Among these, *hsa-miR-138-5p* showed the highest expression in corneal samples and the greatest expression difference between corneal and conjunctival tissues, with a log2 FC of 5.60. Conversely, *hsa-miR-1275* showed the highest expression in conjunctival samples, with a log2 FC of −2.82.

#### 3.1.3. Principal Component Analysis (PCA)

Supplementary Figure S1 shows a clear separation between corneal and conjunctival samples along the first principal component (PC1), which accounted for 40.1% of the total variance, supporting the presence of distinct tissue-specific transcriptomic profiles. Although two corneal samples exhibited greater variation along the second principal component (PC2), they clustered with the remaining corneal samples and did not represent distinct outliers. Therefore, all samples were retained for the downstream analyses.

### 3.2. Target Genes of Differentially Expressed miRNAs

The online platform miRTarLink 2.0 was used to identify target genes of miRNAs showing higher expression in corneal and conjunctival IC samples. Among the 110 miRNAs with higher expression in corneal IC samples, 76 miRNAs were found to regulate a total of 875 target genes. Notably, *hsa-miR-124-3p* exhibited the highest number of target genes, with 88 identified targets. In conjunctival IC samples, 26 of the 65 miRNAs with higher expression were associated with a total of 424 target genes. Among these, *hsa-miR-155-5p* showed the largest number of target genes, regulating 173 targets. Table 4 summarizes the target genes of the top 10 miRNAs showing higher expression in corneal and conjunctival IC samples.

**Figure 2:**
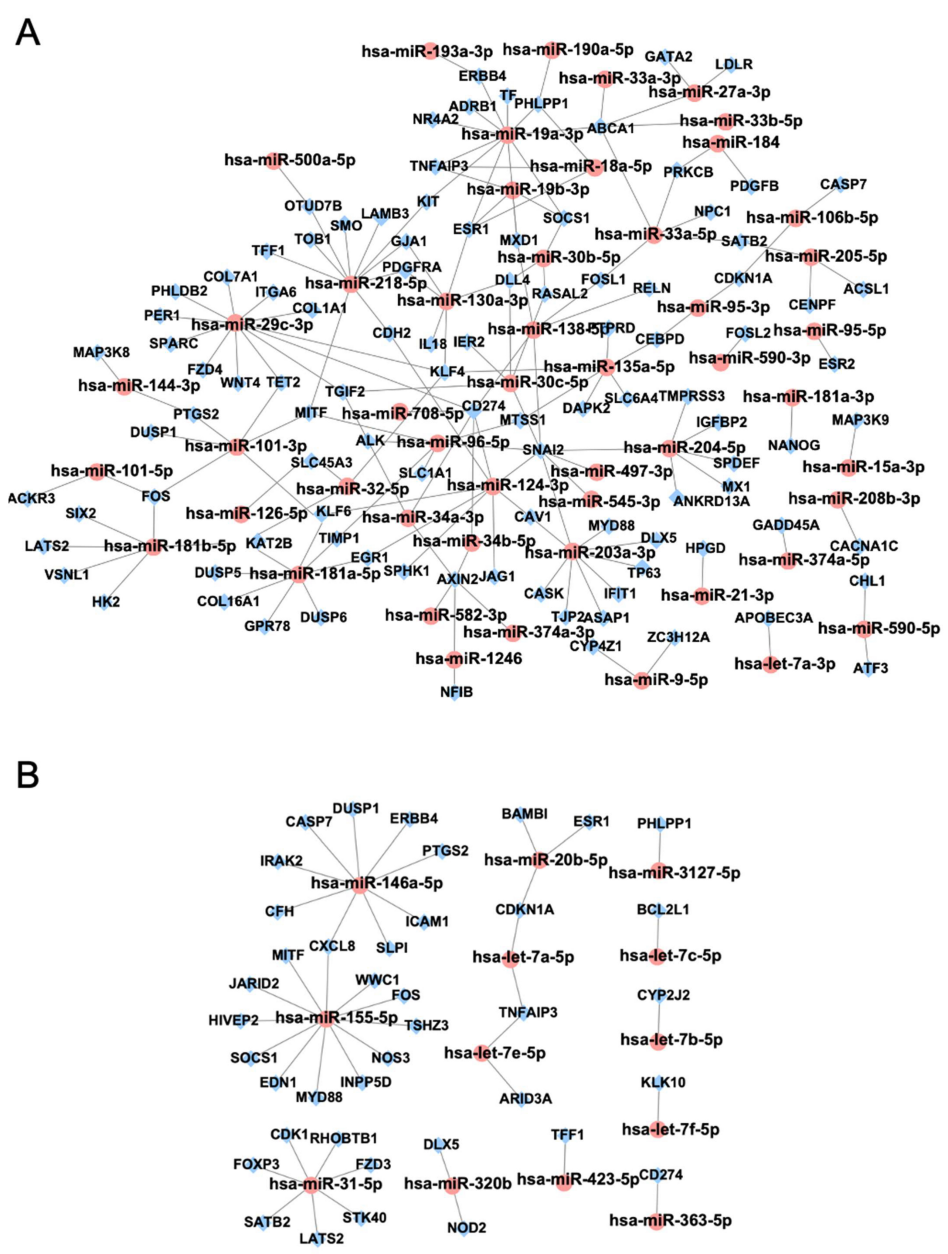
miRNA–mRNA regulatory networks of miRNAs showing higher expression and their target differentially expressed mRNAs in corneal (**A**) and conjunctival (**B**) impression cytology (IC) samples from healthy subjects. Only miRNAs and corresponding target genes that were both differentially expressed between corneal and conjunctival samples were included in the analysis. miRNA–mRNA regulatory interactions were identified using the online platform miRTarLink 2.0 (https://ccb-compute.cs.uni-saarland.de/mirtargetlink2; accessed on 20 April 2026). The miRNAs shown in panel (**A**) exhibit higher expression in corneal IC samples than in conjunctival IC samples, whereas the miRNAs shown in panel (**B**) exhibit higher expression in conjunctival IC samples than in corneal IC samples.

To further investigate the regulatory relevance of these miRNAs, all identified target genes were cross-referenced with the list of DEGs identified between corneal and conjunctival samples. This analysis revealed that 50 miRNAs showing higher expression in the cornea were associated with 105 DEGs, whereas 13 miRNAs showing higher expression in the conjunctiva were linked to 39 DEGs (Figure 2).

### 3.3. PPI Networks and Hub Genes

DEGs showing higher expression in corneal IC samples than in conjunctival IC samples, as well as DEGs showing higher expression in conjunctival IC samples than in corneal IC samples, were analyzed using the STRING database to construct PPI networks.

PPI analysis identified highly interconnected gene networks in both corneal and conjunctival samples (Supplementary Figures S2 and S3).

**Figure 3:**
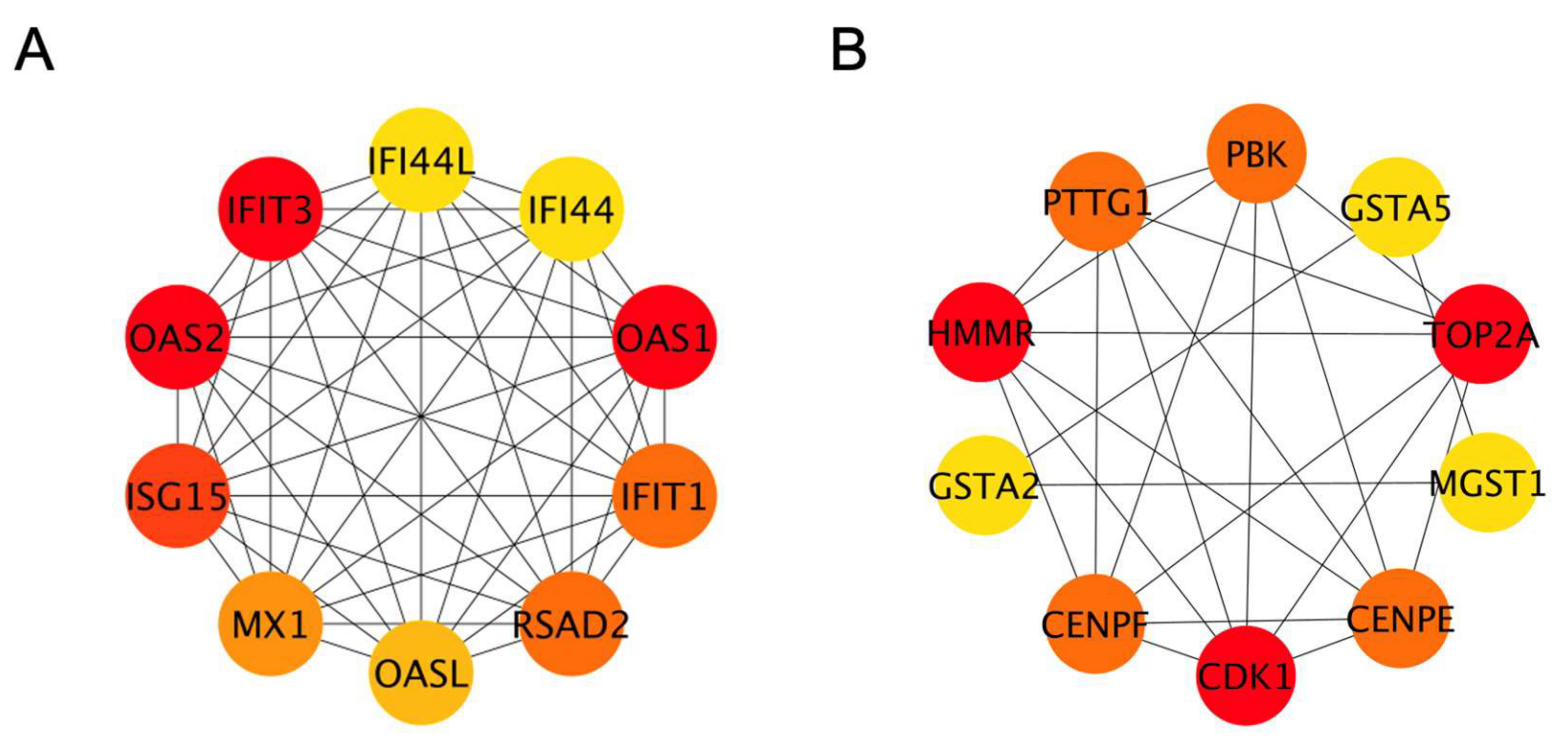
Top 10 hub genes among the differentially expressed genes (DEGs) with higher expression in corneal impression cytology (IC) samples (**A**) and conjunctival IC samples (**B**) from healthy subjects. Color intensity indicates the hub gene ranking generated using the maximal clique centrality (MCC) algorithm.

Hub gene analysis was subsequently performed using MCC. In corneal samples, the top 10 hub genes were *OAS2*, *IFIT3*, *OAS1*, *ISG15*, *RSAD2*, *IFIT1*, *MX1*, *OASL*, *IFI44L*, and *IFI44*. In conjunctival samples, the top 10 hub genes were *CDK1*, *HMMR*, *TOP2A*, *CENPE*, *CENPF*, *PTTG1*, *PBK*, *GSTA5*, *MGST1*, and *GSTA2* (Figure 3).

### 3.4. GO Enrichment Analysis

GO enrichment analysis was performed for DEGs showing higher expression in corneal IC samples than in conjunctival IC samples, as well as for DEGs showing higher expression in conjunctival IC samples than in corneal IC samples. Enriched GO terms were identified across the three GO categories: BP, CC, and MF. Notably, only five MF terms for genes showing higher expression in corneal samples and three MF terms for genes showing higher expression in conjunctival samples reached statistical significance (adjusted *p* value < 0.05).

**Figure 4:**
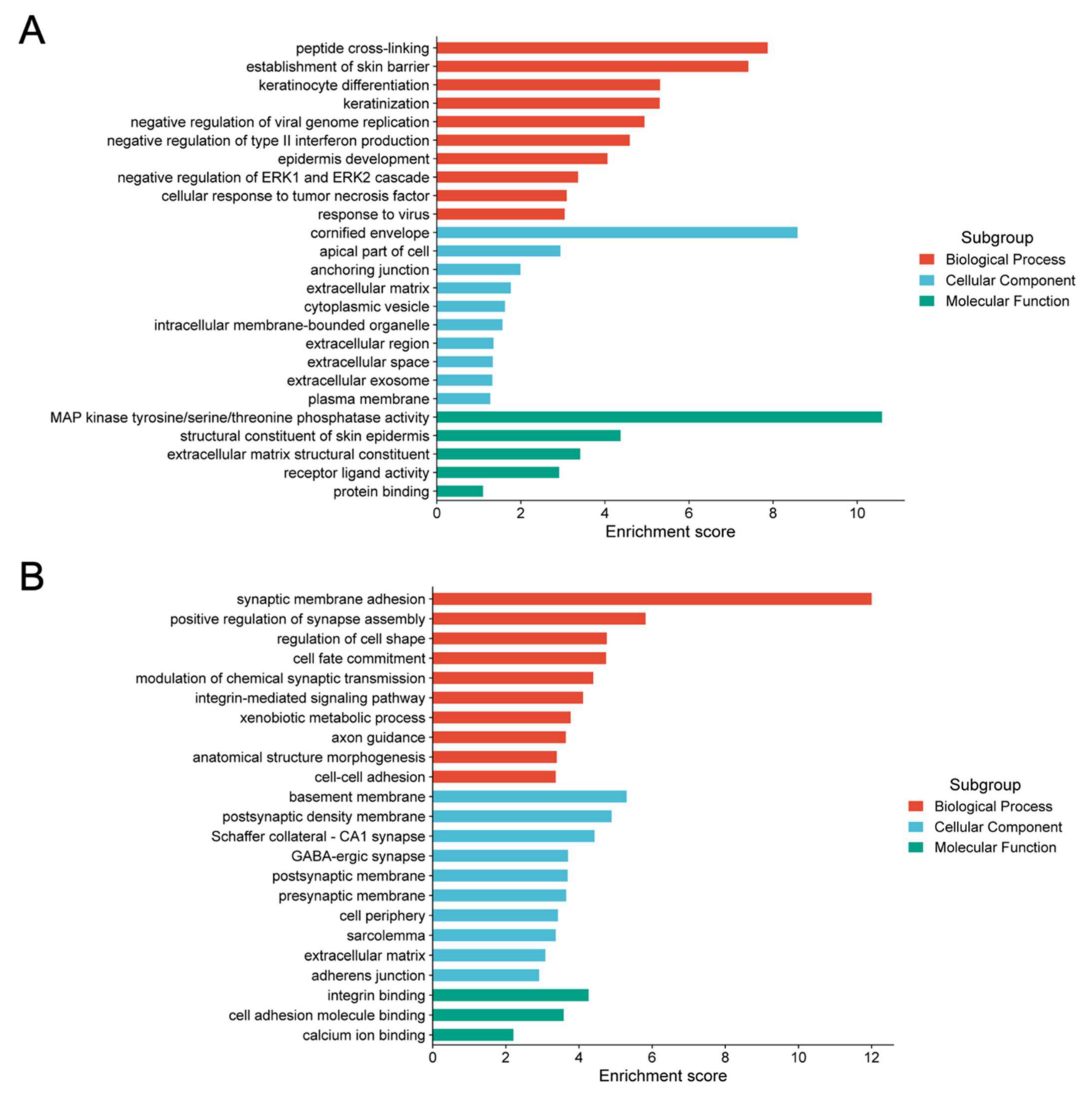
Gene ontology (GO) enrichment terms of mRNAs showing higher expression in corneal (**A**) and conjunctival (**B**) impression cytology (IC) samples from healthy subjects. The X-axis represents the enrichment score, while the Y-axis indicates the corresponding GO terms. For the biological process (BP) and cellular component (CC) categories, the top 10 enriched terms ranked by enrichment score are shown in both panels. For the molecular function (MF) category, all five enriched terms are presented in panel (**A**) and all three enriched terms in panel (**B**).

For DEGs showing higher expression in corneal IC samples, the top 10 enriched BP terms ranked by enrichment score were peptide cross-linking, establishment of skin barrier, keratinocyte differentiation, keratinization, negative regulation of viral genome replication, negative regulation of type II interferon production, epidermis development, negative regulation of the ERK1/ERK2 cascade, cellular response to tumor necrosis factor, and response to virus. In the CC category, the top enriched terms included cornified envelope, apical part of cell, anchoring junction, extracellular matrix, cytoplasmic vesicle, intracellular membrane-bounded organelle, extracellular region, extracellular space, extracellular exosome, and plasma membrane. In the MF category, the significantly enriched terms were MAP kinase tyrosine/serine/threonine phosphatase activity, structural constituent of skin epidermis, extracellular matrix structural constituent, receptor ligand activity, and protein binding (Figure 4A).

For DEGs showing higher expression in conjunctival IC samples, the top 10 enriched BP terms were synaptic membrane adhesion, positive regulation of synapse assembly, regulation of cell shape, cell fate commitment, modulation of chemical synaptic transmission, integrin-mediated signaling pathway, xenobiotic metabolic process, axon guidance, anatomical structure morphogenesis, and cell–cell adhesion. In the CC category, the top enriched terms included basement membrane, postsynaptic density membrane, Schaffer collateral-CA1 synapse, GABAergic synapse, postsynaptic membrane, presynaptic membrane, cell periphery, sarcolemma, extracellular matrix, and adherens junction. In the MF category, the significantly enriched terms were integrin binding, cell adhesion molecule binding, and calcium ion binding (Figure 4B).

### 3.5. KEGG Pathway Analysis

**Figure 5:**
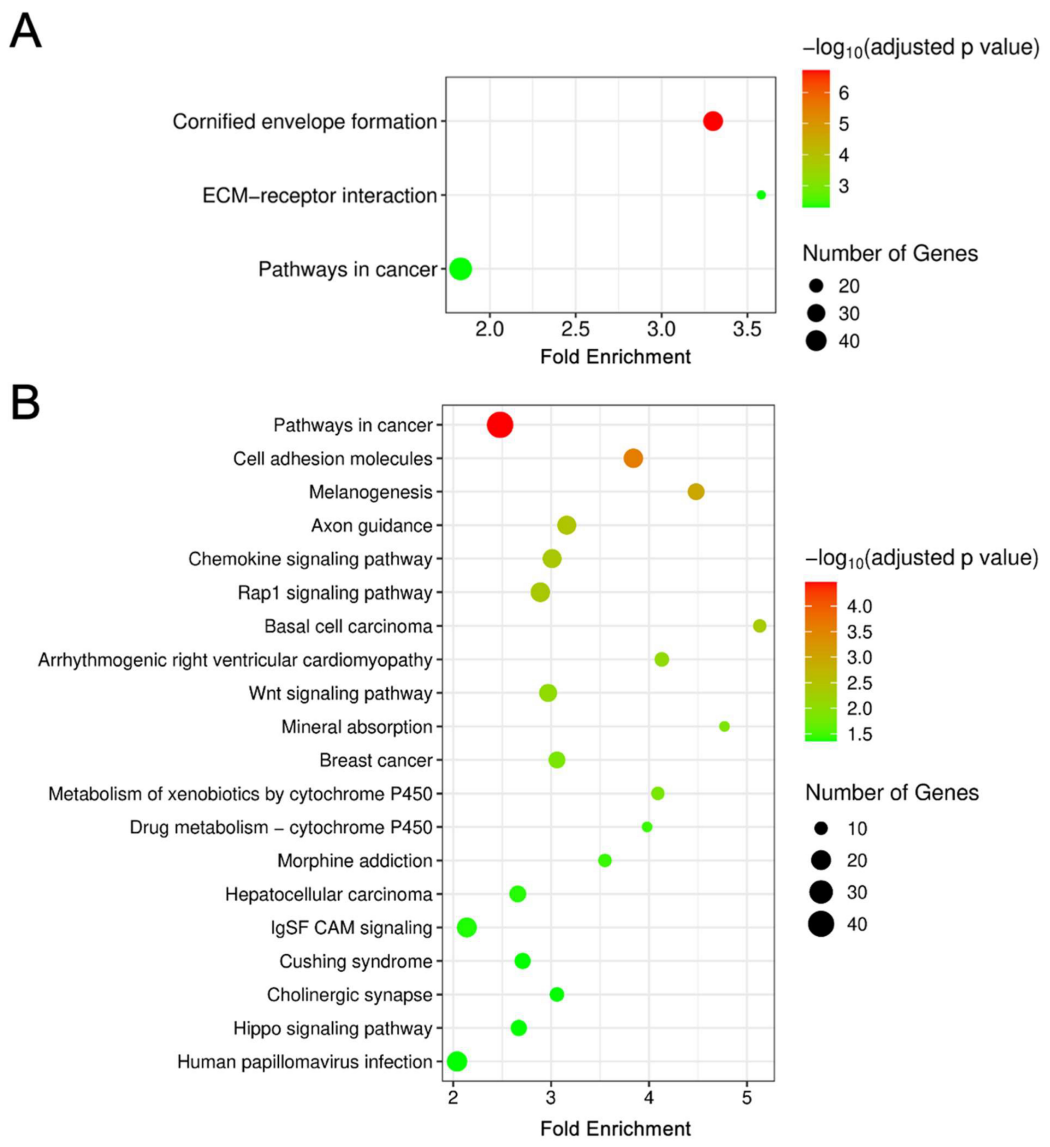
Bubble plots of Kyoto encyclopedia of genes and genomes (KEGG) pathway enrichment analysis of mRNAs showing higher expression in corneal (**A**) and conjunctival (**B**) impression cytology (IC) samples from healthy subjects. In the bubble plots, the size of each circle represents the number of genes associated with the corresponding pathway, whereas the color indicates the adjusted *p* value, reflecting the statistical significance of pathway enrichment. The X-axis represents fold enrichment, and the Y-axis shows the corresponding KEGG pathways.

Using the same set of DEGs, we subsequently performed KEGG pathway enrichment analysis. Among the DEGs showing higher expression in corneal samples, three significantly enriched pathways were identified: cornified envelope formation, ECM-receptor interaction, and pathways in cancer (Figure 5A).

In contrast, 20 significantly enriched pathways were identified among the DEGs showing higher expression in conjunctival samples. These included pathways in cancer, cell adhesion molecules, melanogenesis, axon guidance, chemokine signaling pathway, Rap1 signaling pathway, basal cell carcinoma, arrhythmogenic right ventricular cardiomyopathy, Wnt signaling pathway, mineral absorption, metabolism of xenobiotics by cytochrome P450, breast cancer, drug metabolism–cytochrome P450, morphine addiction, hepatocellular carcinoma, IgSF CAM signaling, cholinergic synapse, Cushing syndrome, Hippo signaling pathway, and human papillomavirus infection (Figure 5B).

### 3.6. RT-qPCR Validation

**Figure 6:**
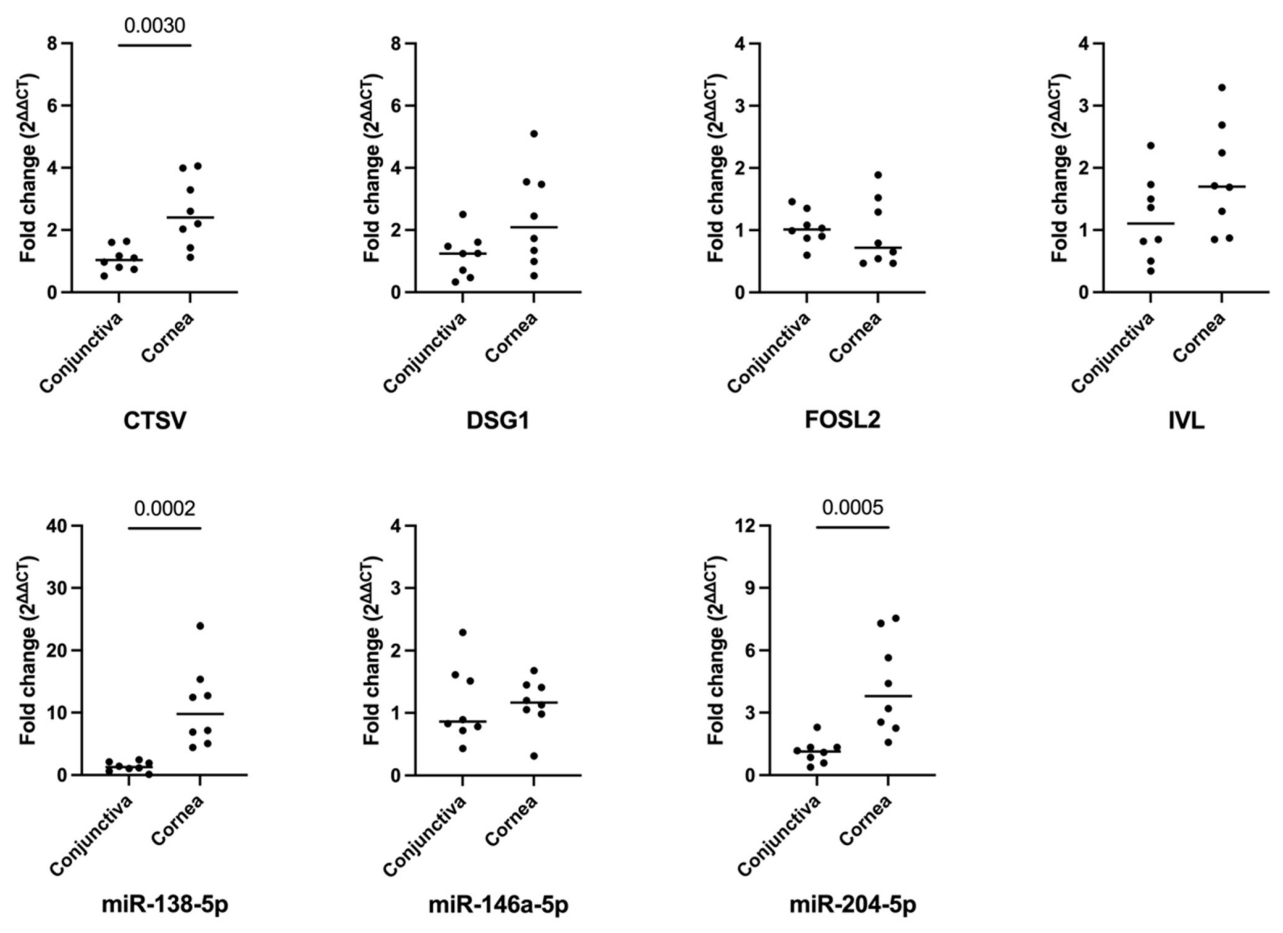
RT-qPCR validation results of selected mRNA and miRNA expression levels in corneal and conjunctival impression cytology (IC) samples from healthy subjects (*n* = 8). Expression levels were quantified by RT-qPCR and are presented as 2ΔΔCT fold changes. Statistical comparisons between corneal and conjunctival samples were performed using the Mann–Whitney test. CTSV, miR-138-5p, and miR-204-5p were significantly upregulated in corneal samples compared with conjunctival samples (*p* = 0.0030, *p* = 0.0002, and *p* = 0.0005, respectively). In contrast, no statistically significant differences were observed for the remaining mRNAs and miRNAs (*p* ≥ 0.1049).

To validate the differential expression profiles identified by sequencing, selected mRNAs and miRNAs from corneal and conjunctival IC samples were further analyzed by RT-qPCR. The selected mRNAs included *CTSV* (FC = 8.66, *p* < 0.0001), *DSG1* (FC = 8.12, *p* < 0.0001), *FOSL2* (FC = 2.01, *p* < 0.0001), and *IVL* (FC = 4.38, *p* < 0.0001). The selected miRNAs included *hsa-miR-138-5p* (FC = 48.52, *p* < 0.0001), *hsa-miR-146a-5p* (FC = 0.17, *p* = 0.0033), and *hsa-miR-204-5p* (FC = 5.21, *p* = 0.0015). RT-qPCR validation demonstrated that *CTSV*, *hsa-miR-138-5p*, and *hsa-miR-204-5p* showed significantly higher expression in corneal IC samples than in conjunctival IC samples (*p* = 0.0030, *p* = 0.0002, and *p* = 0.0005, respectively). In contrast, no statistically significant differences were observed for the remaining mRNAs and miRNAs (*p* ≥ 0.1049) (Figure 6).

## 4. Discussion

In the present study, we systematically characterized the mRNA and miRNA expression profiles of healthy human corneal and conjunctival epithelial cells using IC coupled with RNA sequencing and integrated bioinformatic analyses. Our results revealed distinct tissue-specific molecular signatures between the two ocular surface epithelia. Compared with the conjunctiva, the cornea exhibited a transcriptomic profile predominantly associated with epithelial differentiation, barrier maintenance, tissue renewal, and antiviral defense, whereas the conjunctiva showed enrichment of genes involved in immune regulation, tissue remodeling, and metabolic detoxification. Integrated analyses of differentially expressed genes, miRNAs, functional enrichment, and protein–protein interaction networks consistently reflect these tissue-specific molecular characteristics, providing a comprehensive reference for understanding the physiological differences between the corneal and conjunctival epithelia.

### 4.1. mRNA and miRNA Expression Profiles and Analysis in the Cornea

Compared with the conjunctiva, the cornea exhibited higher expression of genes associated with epithelial differentiation, structural integrity, tissue repair, and innate immune defense. Among the most highly expressed genes, *KRT24*, *KRT16*, together with the well-established corneal markers *KRT3* and *KRT12*, reflects the highly differentiated phenotype of the corneal epithelium [30,31]. In addition, genes involved in epithelial barrier formation and extracellular matrix organization, including *SPRR2D*, *ECM1*, *KLK7*, and *TMPRSS11B*, were also enriched in corneal samples [32,33,34,35]. Higher expression of *IL36RN*, *RNASE7*, and *HSPB8* further suggests enhanced antimicrobial and immune regulatory capacity [36,37,38], whereas *EREG*, *AREG*, and *MYEOV* are associated with epithelial renewal and wound repair [39,40,41]. Furthermore, the increased expression of *MUC22*, *MUCL3*, and *SFTA2* may contribute to tear film stability and maintenance of the ocular surface microenvironment [42,43]. Collectively, these findings indicate that the corneal epithelium possesses a molecular profile consistent with its specialized barrier function and continuous exposure to the external environment.

These tissue-specific characteristics were consistently supported by the integrated functional enrichment analyses. GO and KEGG analyses demonstrated enrichment of biological processes related to epithelial differentiation, keratinization, epidermal development, extracellular matrix organization, and barrier formation. Rather than representing the complete physiological activities of the cornea, these enriched pathways reflect the molecular features that distinguish the corneal epithelium from the conjunctival epithelium. In addition, genes associated with antiviral responses were significantly enriched, including pathways involved in viral defense and interferon-mediated immune responses. As the outermost layer of the eye, the corneal epithelium serves as the first barrier against environmental pathogens, particularly viral infections such as herpes simplex keratitis [44,45]. Therefore, the enrichment of antiviral pathways suggests that healthy corneal epithelial cells maintain a transcriptional profile compatible with rapid innate immune responses while preserving immune homeostasis. Enrichment of MAPK/ERK-related pathways is also consistent with the established role of this signaling pathway in regulating corneal epithelial proliferation, migration, and differentiation [46]. Notably, several miRNAs showing higher expression in the cornea in our study, including miR-138-5p, miR-135a-5p, and miR-204-5p, etc., have previously been reported to regulate MAPK/ERK-related pathways [47,48,49].

It has been estimated that approximately 30% of protein-coding genes are regulated by miRNAs [50], and disruption of the normal miRNA–mRNA regulatory network can lead to profound cascading biological effects [51]. In our study, miRNAs exhibited expression patterns consistent with those of mRNAs, with both the number and magnitude of miRNAs showing higher expression being greater in the cornea than in the conjunctiva. Among them, miR-138-5p showed the highest expression in the cornea, which was further validated by our RT-qPCR results. Previous studies have demonstrated that miR-138-5p is significantly upregulated in conditions such as keratoconus and aniridia-associated keratopathy [47,52]. Its target genes are widely involved in key signaling pathways, including PI3K-Akt, MAPK, and JAK-STAT pathways [47]. In addition, overexpression of miR-138-5p has been reported to inhibit proliferation and induce cell cycle arrest in limbal epithelial cells [53]. miRNAs also play important roles in various physiological and pathological processes of the cornea. For example, miR-126 and miR-204-5p are associated with corneal neovascularization [15]; miR-182, miR-29, and miR-21 are implicated in herpes simplex keratitis [54]; miR-27a-3p regulates inflammation and neurodegeneration in diabetic corneas [55]; and miR-184 has been shown to promote corneal wound healing [56]. Notably, these miRNAs were all found to be highly expressed in the cornea in our dataset. Based on these findings, we constructed a miRNA–mRNA regulatory network focusing on miRNAs and mRNAs showing higher expression in the cornea (Figure 2A). This network provides a more intuitive understanding of the interactions between miRNAs and their target genes and may offer valuable insights for future research directions as well as potential therapeutic targets for corneal diseases.

Further support for these findings was provided by the hub gene analysis. All ten hub genes identified in the corneal protein–protein interaction network belonged to interferon-stimulated genes (ISGs), which are widely recognized as key mediators of innate antiviral immunity [57,58]. Their coordinated higher expression is consistent with the enrichment of antiviral pathways identified by GO and KEGG analyses and suggests that the healthy corneal epithelium maintains a transcriptional state that enables rapid responses to viral challenges while avoiding excessive inflammation [6,59]. Taken together, the integrated transcriptomic and bioinformatic analyses consistently indicate that the corneal epithelium is characterized by molecular features associated with epithelial integrity, barrier function, tissue renewal, and innate immune defense.

### 4.2. mRNA and miRNA Expression Profiles and Analysis in the Conjunctiva

In contrast, the mRNA and miRNA expression profiles of the conjunctiva, as well as their corresponding analyses, exhibited distinct characteristics. Compared with the cornea, fewer genes and miRNAs showed higher expression in the conjunctiva. However, these genes displayed greater functional diversity. The genes showing the highest expression in the conjunctiva were mainly involved in immune response and secretion, metabolic detoxification, extracellular matrix remodeling, and key signaling pathway regulation. For example, LTF, which encodes lactoferrin, is an important antimicrobial protein in tears and is also involved in anti-inflammatory responses [60]. *SCGB3A1* and *FDCSP*, both secreted proteins, are associated with immune regulation [61,62]. In addition, *APELA* may function as a chemokine regulating angiogenic cells, suggesting that, compared with the cornea, the conjunctiva possesses stronger vascularization and immune regulatory capacity [63]. Collectively, these genes indicate that the conjunctiva plays a crucial role in maintaining the immune barrier of the ocular surface through the secretion of protective proteins. Second, regarding metabolic and detoxification functions, *GSTA1* and *GSTA2* belong to the glutathione S-transferase (GST) family and are involved in oxidative stress responses and the metabolism of xenobiotics [64]. Furthermore, previous studies have reported significantly increased GST expression in patients with pterygium, suggesting that the conjunctiva possesses strong antioxidant capacity in response to environmental stimuli such as ultraviolet radiation and pharmacological exposure [65]. In terms of extracellular matrix remodeling and tissue renewal, MMP1, a matrix metalloproteinase, is involved in extracellular matrix degradation and remodeling [66]; *SPARCL1*, *PTN*, and *CTNNA3* are associated with cell adhesion, proliferation, migration, differentiation, and tissue repair [67,68,69]. In addition, *CA12* plays an important role in regulating local pH and ion balance [70]. Together, these genes contribute to maintaining the stability of the extracellular environment in the conjunctiva.

These observations were further supported by the integrated functional enrichment analyses. GO and KEGG analyses consistently demonstrated enrichment of pathways related to immune regulation, cell adhesion, extracellular matrix organization, tissue remodeling, signal transduction, and xenobiotic metabolism. Compared with the cornea, the conjunctiva exhibited broader enrichment of pathways associated with environmental adaptation and epithelial plasticity. Enrichment of synapse-related terms and axon guidance pathways is consistent with the rich sensory innervation of the conjunctiva and may reflect molecular characteristics associated with ocular surface sensation and tear reflex regulation [71,72]. Likewise, enrichment of chemokine signaling pathways supports the established role of the conjunctiva in coordinating immune surveillance at the ocular surface. The enrichment of cytochrome P450-related metabolic pathways further suggests enhanced capacity for detoxification of environmental agents, which is consistent with the continuous exposure of the conjunctival surface to airborne particles, microorganisms, ultraviolet radiation, and topical medications [73].

Similarly, the number of miRNAs showing higher expression in the conjunctiva was also lower than that in the cornea, showing a consistent trend with mRNA expression. Among the miRNAs showing higher expression in the conjunctiva in our study, miR-146a and miR-155 have been associated with conjunctivitis [74], miR-642a-5p with pterygium [75], and miR-31 with Stevens–Johnson syndrome [76]. Furthermore, we constructed a regulatory network of miRNAs and mRNAs showing higher expression in the conjunctiva (Figure 2B), providing a comprehensive overview of their interactions.

Hub gene analysis further supported these observations. The top ten hub genes identified in the conjunctival protein–protein interaction network could be broadly classified into genes associated with cell cycle regulation (*CDK1*, *TOP2A*, *CENPE*, *CENPF*, *PTTG1*, *PBK*, and *HMMR*) and metabolic detoxification (*GSTA2*, *GSTA5*, and *MGST1*) [77,78,79,80,81,82,83,84]. Compared with the cornea, the conjunctival epithelium exhibits molecular characteristics consistent with active epithelial turnover and enhanced adaptation to environmental challenges. Together, the integrated transcriptomic and bioinformatic analyses indicate that the conjunctiva is distinguished from the cornea by molecular features associated with immune regulation, tissue remodeling, and metabolic detoxification.

Furthermore, although RT-qPCR confirmed the differential expression of three of the seven selected targets, the remaining targets did not reach statistical significance. This partial validation may reflect the relatively small validation cohort, inter-individual biological variability, and inherent methodological differences between RNA sequencing and RT-qPCR. Therefore, further validation in larger, independent cohorts will be important to confirm the robustness and reproducibility of these findings.

### 4.3. Limitations

Although this study provides a comprehensive comparison of the mRNA and miRNA expression profiles of healthy human corneal and conjunctival epithelial cells using impression cytology and integrated bioinformatic analyses, several limitations should be acknowledged. First, the sample size was relatively small, which may have limited the statistical power for detecting subtle transcriptional differences. Validation in larger independent cohorts will therefore be important to improve the robustness and generalizability of these findings. Second, although no statistically significant differences in age or sex distribution were observed between the corneal and conjunctival cohorts, the corneal group included a wider age range, whereas all conjunctival samples were obtained from adults. Therefore, the potential influence of age-related transcriptional variation cannot be completely excluded. Future studies using larger, age-matched cohorts and incorporating age and sex as covariates in differential expression analyses will be important to further validate the tissue-specific molecular signatures identified in this study. Third, impression cytology primarily samples superficial epithelial cells and therefore does not fully represent deeper epithelial layers or the underlying stromal tissue. Finally, the biological implications discussed in the present study are primarily based on transcriptomic and bioinformatic analyses. Although selected genes and miRNAs were validated by RT-qPCR, additional functional studies are required to confirm the biological roles of the identified genes, miRNAs, and regulatory networks.

## 5. Conclusions

This study systematically analyzed the mRNA and miRNA expression profiles of the normal human cornea and conjunctiva based on impression cytology samples, and comprehensively explored their molecular characteristics and miRNA–mRNA regulatory networks using multiple bioinformatics approaches. In addition to their shared involvement in maintaining epithelial structure, tissue renewal, and immune responses, the cornea displays highly specialized gene expression features related to antiviral defense, reflecting its robust barrier function. In contrast, the conjunctiva is more prominently associated with metabolic detoxification and antioxidant processes, enabling it to effectively cope with environmental stressors. In summary, this study reveals both the molecular differences and cooperative roles of the cornea and conjunctiva, deepens our understanding of ocular surface physiology and its miRNA–mRNA regulatory mechanisms, and provides valuable references for future research into the pathogenesis and clinical management of ocular surface diseases.

## Figures and Tables

**Table 1:** Demographic characteristics of corneal and conjunctival impression cytology (IC) samples ^1^.

Corneal Samples			Conjunctival Samples		
Sample No.	Gender	Age (Years)	Sample No.	Gender	Age (Years)
1	Male	2	1	Female	22
2	Male	6	2	Male	25
3	Male	9	3	Male	26
4	Female	16	4	Female	27
5	Male	18	5	Male	27
6	Female	19	6	Male	27
7	Female	22	7	Female	29
8	Female	23	8	Male	33
9	Male	35	9	Female	39
10	Female	42	10	Female	60
11	Female	49			
12	Female	49			
13	Male	59			
14	Female	60			
Total: 14 samples	7 males 7 females	29.0 ± 19.4	Total: 10 samples	5 males 5 females	31.5 ± 11.1

^1^ All corneal and conjunctival samples were obtained from different individuals.

**Table 2:** Top 20 differentially expressed genes (DEGs) showing higher expression in corneal or conjunctival impression cytology (IC) samples.

Higher Expression in Corneal IC Samples			Higher Expression in Conjunctival IC Samples		
Gene	Log2 FC	Adjusted *p* Value	Gene	Log2 FC	Adjusted *p* Value
*KRT24*	9.08	<0.0001	*APELA*	−3.63	<0.0001
*MYEOV*	9.00	<0.0001	*LTF*	−3.51	0.0079
*KRT12*	8.78	<0.0001	*GSTA1*	−3.38	0.0002
*ECM1*	8.25	<0.0001	*SPARCL1*	−3.33	0.0003
*MUC22*	7.67	<0.0001	*MYBPC1*	−3.32	0.0005
*IL36RN*	7.63	<0.0001	*CTNNA3*	−3.28	<0.0001
*KLK7*	7.16	<0.0001	*CA12*	−3.25	<0.0001
*EREG*	7.11	<0.0001	*SCGB3A1*	−3.20	0.0008
*KRT16*	6.81	<0.0001	*GSTA2*	−3.15	0.0038
*RNASE7*	6.20	<0.0001	*ANOS1*	−3.15	<0.0001
*MAL*	6.15	<0.0001	*PTN*	−3.12	0.0036
*TMPRSS11B*	6.09	<0.0001	*MMP1*	−3.11	<0.0001
*KRT3*	5.99	<0.0001	*HEYL*	−3.11	0.0001
*AREG*	5.92	<0.0001	*PEG10*	−3.06	0.0010
*SPRR2D*	5.92	<0.0001	*STEAP2*	−3.06	0.0048
*EFHD1*	5.88	<0.0001	*NALF1*	−2.93	<0.0001
*ANKFN1*	5.83	<0.0001	*GRIA4*	−2.92	<0.0001
*SFTA2*	5.81	<0.0001	*FDCSP*	−2.91	0.0013
*MUCL3*	5.78	<0.0001	*HEY2*	−2.91	<0.0001
*HSPB8*	5.48	<0.0001	*WSCD2*	−2.89	0.0007

**Table 3:** Top 20 differentially expressed miRNAs showing higher expression in corneal or conjunctival impression cytology (IC) samples.

Higher Expression in Corneal IC Samples			Higher Expression in Conjunctival IC Samples		
miRNA	Log2 FC	Adjusted *p* Value	miRNA	Log2 FC	Adjusted *p* Value
*hsa-miR-138-5p*	5.60	<0.0001	*hsa-miR-1275*	−2.82	0.0025
*hsa-miR-451a*	5.59	0.0066	*hsa-miR-146a-5p*	−2.55	0.0033
*hsa-miR-135a-5p*	4.94	<0.0001	*hsa-miR-877-5p*	−2.37	0.0064
*hsa-miR-708-5p*	4.48	<0.0001	*hsa-miR-375-3p*	−2.32	0.0007
*hsa-miR-144-3p*	4.16	0.0176	*hsa-miR-664b-5p*	−2.21	0.0033
*hsa-miR-181a-3p*	4.03	<0.0001	*hsa-miR-31-5p*	−2.20	0.0004
*hsa-miR-193a-3p*	3.29	<0.0001	*hsa-miR-10395-3p*	−2.16	0.0015
*hsa-miR-218-5p*	2.99	0.0006	*hsa-miR-342-5p*	−2.13	0.0044
*hsa-miR-139-5p*	2.95	0.0004	*hsa-miR-92b-5p*	−2.02	0.0018
*hsa-miR-708-3p*	2.93	<0.0001	*hsa-miR-760*	−1.88	0.0018
*hsa-miR-4454*	2.82	0.0004	*hsa-miR-155-5p*	−1.85	0.0426
*hsa-miR-3182*	2.70	0.0003	*hsa-miR-320a-3p*	−1.78	0.0439
*hsa-miR-33b-5p*	2.44	<0.0001	*hsa-miR-92a-2-5p*	−1.74	0.0030
*hsa-miR-204-5p*	2.38	0.0015	*hsa-miR-664a-5p*	−1.74	0.0055
*hsa-miR-362-3p*	2.35	0.0003	*hsa-let-7b-5p*	−1.74	0.0037
*hsa-miR-33a-5p*	2.30	<0.0001	*hsa-miR-1262*	−1.73	0.0059
*hsa-miR-1290*	2.29	0.0019	*hsa-let-7c-5p*	−1.72	0.0012
*hsa-miR-188-5p*	2.28	0.0001	*hsa-miR-3127-5p*	−1.71	0.0146
*hsa-miR-181a-5p*	2.12	0.0001	*hsa-miR-4446-3p*	−1.68	0.0189
*hsa-miR-33a-3p*	2.10	0.0001	*hsa-miR-505-5p*	−1.68	0.0103

**Table 4:** Target genes of the top 10 miRNAs showing higher expression in corneal or conjunctival impression cytology (IC) samples.

miRNA	Target Genes
**miRNAs showing higher expression in corneal IC samples**	
hsa-miR-138-5p	*ROCK2*, *SNAI2*, *BLCAP*, *H2AFX*, *RELN*, *VIM*, *EZH2*, *TERT*, *RMND5A*, *CD274*, *AKT1*, *NFKB1*, *ADGRA2*, *SOX9*, *S100A1*, *CDH1*, *KDM5C*, *BAG1*, *HIF1A*, *CYTOR*, *CASP3*, *YAP1*, *BCL11A*, *EIF4EBP1*, *SIRT1*, *MXD1*, *RHOC*, *FOSL1*, *SOX4*, *MAP3K11*, *PTK2*, *RARA*, *FOXC1*, *LCN2*, *FERMT2*, *SENP1*, *CCND3*, *ZEB2*, *GNAI2*, *EID1*, *SUZ12*, *CCND1*, *TWIST2*
hsa-miR-451a	*MYC*, *IKBKB*, *RAB5A*, *MIF*, *CDKN2D*, *DCBLD2*, *TMED7*, *ABCB1*, *CAB39*, *MMP2*, *MMP9*, *AKT1*, *RAB14*, *IL6R*, *OXTR*, *CPNE3*, *IL6*, *MAPK1*, *BCL2*, *ADAM10*, *MAP3K1*, *TSC1*
hsa-miR-135a-5p	*VLDLR*, *ROCK2*, *MYC*, *APC*, *CEBPD*, *NR3C2*, *BMPR2*, *EGFR*, *FOXO1*, *PPM1E*, *PTPRD*, *TXNIP*, *DAPK2*, *E2F1*, *MMP11*, *KLF8*, *JAK2*, *RUNX2*, *IRS2*, *SLC6A4*, *PHLPP2*, *MTSS1*, *PTK2*, *RBAK*, *HOXA10*, *HTR1A*, *SMAD5*, *SIAH1*, *BCL2*, *KLF4*, *STAT6*, *ROCK1*, *ESRRA*
hsa-miR-708-5p	*CNTFR*, *TMEM88*, *PARP1*, *BIRC5*, *CD44*, *AKT2*, *SMAD3*, *EZH2*, *MMP2*, *BMI1*, *CD274*, *AKT1*, *NNAT*, *EYA3*, *CASP2*, *BCL2*, *KDM1A*, *ZEB2*, *IKBKG*, *CCND1*
hsa-miR-144-3p	*NFE2L2*, *ZEB1*, *CFTR*, *TTN*, *PLAG1*, *XIST*, *FGG*, *EZH2*, *ETS1*, *MET*, *SMAD4*, *NOTCH1*, *TUG1*, *TGFB1*, *Adamts1*, *IRS1*, *PBX3*, *MAP3K8*, *APP*, *MTOR*, *PTGS2*, *ZEB2*, *PTEN*
hsa-miR-181a-3p	*NANOG*
hsa-miR-193a-3p	*HMGB1*, *E2F6*, *KRAS*, *RPS6KB2*, *MCL1*, *SELENON*, *E2F1*, *SRSF2*, *MMP14*, *ERBB2*, *ERBB4*, *TGFB2*, *PRAP1*, *PLAU*, *SLC7A5*, *PTEN*, *HYOU1*, *TYMS*, *CCND1*, *RAB27B*
hsa-miR-218-5p	*BIRC5*, *IKBKB*, *POU2F2*, *OTUD7B*, *VPS51*, *RPS6KB1*, *ROBO1*, *DKK2*, *MITF*, *EGFR*, *SH3GL1*, *BCL9*, *SMO*, *RPS6KA3*, *PDGFRA*, *LEF1*, *VOPP1*, *RET*, *SP1*, *E2F2*, *WASF3*, *HMGB1*, *CDH2*, *RICTOR*, *KIT*, *RUNX2*, *TOB1*, *HMOX1*, *HOXB3*, *GLI2*, *LAMB3*, *MMP2*, *LASP1*, *BMI1*, *CDK6*, *GJA1*, *TFF1*, *SOST*, *SFRP2*, *LGR4*
hsa-miR-139-5p	*ROCK2*, *MMP11*, *FAM162A*, *MCL1*, *NR5A2*, *SMARCA4*, *RHOT1*, *ZHX2*, *TPD52*, *WNT1*, *OIP5*, *MET*, *PDE4D*, *HRAS*, *NOTCH1*, *IGF1R*, *NFKB1*, *CXCR4*, *RAP1B*, *BCL2*, *ADGRL4*, *JUN*, *FOS*, *ACTC1*, *PIK3CA*
hsa-miR-708-3p	*TEX261*, *OTUB*, *N4BP1*, *MPL*
**miRNAs showing higher expression in conjunctival IC samples**	
hsa-miR-146a-5p	*COPS8*, *TLR4*, *COX2*, *CCL5*, *CXCL12*, *LFNG*, *NFKB1*, *PLAUR*, *FADD*, *LAMC2*, *NUMB*, *UHRF1*, *IRAK1*, *IRAK2*, *MTA2*, *NOS1*, *ELAVL1*, *NOTCH2*, *RHOA*, *PRKCE*, *CD80*, *WASF2*, *TGFB1*, *TRAF6*, *CARD10*, *SOS1*, *PTGS2*, *ROCK1*, *FANCM*, *CASP7*, *CCND1*, *NFAT5*, *RHO*, *BRCA1*, *BRCA2*, *RARB*, *BCLAF1*, *EGFR*, *CNOT6L*, *CCND2*, *SMAD4*, *SMN1*, *RNF11*, *CCNA2*, *ERBB4*, *IL6*, *CXCR4*, *CXCL8*, *CPM*, *DUSP1*, *PA2G4*, *FAS*, *SLPI*, *STAT1*, *MIF*, *CD40LG*, *LRP2*, *FAF1*, *SMAD2*, *PTGES2*, *CFH*, *NOTCH1*, *RAC1*, *SOX2*, *L1CAM*, *IS2*, *SIKE1*, *TLR2*, *HOXD10*, *ICAM1*
hsa-miR-664b-5p	*CCNE2*
hsa-miR-31-5p	*MMP16*, *MET*, *SPRY1*, *SPRY4*, *STK40*, *BAP1*, *SELE*, *XRCC5*, *RHOBTB1*, *RASA1*, *MAP4K4*, *DOCK1*, *HIF1AN*, *TBXA2R*, *PPP2R2A*, *DMD*, *ARID1A*, *SRC*, *CASR*, *SPRED2*, *EMSY*, *LATS2*, *CREG1*, *MLH1*, *SPRED1*, *ABCB9*, *RDX*, *SATB2*, *SMAD4*, *MPRIP*, *DKK1*, *IL25*, *GNA13*, *MCM2*, *DACT3*, *SGPP2*, *SP7*, *FZD3*, *STMN1*, *E2F2*, *RAB27A*, *WASF3*, *ITGA5*, *TIAM1*, *CDK1*, *SPRY3*, *FOXP3*, *C1QTNF9*, *FOXO3*, *SOX4*, *SLC1A2*, *RHOA*, *PRKCE*
hsa-miR-342-5p	*ENG*, *NAA10*
hsa-miR-760	*HIST1H3D*, *HIST1H2AD*, *CSNK2A1*
hsa-miR-155-5p	*LCORL*, *MSH2*, *AGO4*, *UQCRFS1*, *NSD3*, *MAP3K10*, *TAF5L*, *IKBKE*, *LNX2*, *BCL6*, *SMARCA4*, *PKIA*, *APAF1*, *PHC2*, *VHL*, *ZNF28*, *MBNL3*, *NOS3*, *FGF7*, *HIF1A*, *FAM199X*, *MSH6*, *STAT1*, *ARMC2*, *MAPK14*, *SKI*, *NR1H3*, *TFAM*, *RUNX2*, *ZNF254*, *FOXO3*, *ANAPC16*, *TRAK1*, *FBXW7*, *SMAD2*, *MATR3*, *PCDH9*, *CKAP5*, *ZIC3*, *SMAD5*, *ARID2*, *GATM*, *PKN2*, *RAD51*, *KDM3A*, *TBC1D14*, *RAPGEF2*, *SDCBP*, *APC*, *MAP3K14*, *BACH1*, *RPTOR*, *MITF*, *TRIP13*, *NFKB1*, *FADD*, *PALD1*, *E2F2*, *SAMHD1*, *IL13RA1*, *SOCS1*, *PSIP1*, *MYO10*, *CLUAP1*, *TBRG1*, *CEP83*, *SEL1L*, *ETS1*, *OLR1*, *WWC1*, *HOMEZ*, *MXI1*, *ANXA2*, *C3orf18*, *FAM177A1*, *CYR61*, *MRPL18*, *MCM8*, *MYBL1*, *IL17RB*, *KLHL5*, *ARL15*, *TAB2*, *CCND1*, *TRIM32*, *CSNK1A1*, *TLE4*, *MEF2A*, *MLH1*, *CD68*, *MYD88*, *LRIF1*, *CCND2*, *JARID2*, *NARS*, *IFNGR1*, *CEP41*, *INPP5D*, *LDOC1*, *CXCL8*, *RNF123*, *CASP3*, *GCSAM*, *MASTL*, *SECISBP2*, *TERF1*, *DCUN1D2*, *INTS6*, *TM6SF1*, *EXOSC2*, *MECP2*, *RAB11FIP2*, *SHANK2*, *MPP5*, *C16orf62*, *CYP2U1*, *CSF1R*, *CARHSP1*, *TSHZ3*, *BRPF3*, *VPS18*, *MAFB*, *FOS*, *PIK3R1*, *PTEN*, *DHX40*, *WEE1*, *MAPK13*, *MYLK*, *MYC*, *MORC3*, *MEIS1*, *PRKAR1A*, *PHF14*, *GPM6B*, *PAK2*, *PICALM*, *PDLIM5*, *WBP1L*, *EDN1*, *TSPAN14*, *SAP30L*, *SPI1*, *CARD11*, *MARF1*, *RHOA*, *SOCS6*, *MMP16*, *ZNF611*, *ZNF652*, *MYB*, *UBQLN1*, *SMAD1*, *HBP1*, *CLDN1*, *FAM91A1*, *FAM135A*, *THRB*, *KBTBD2*, *TP53INP1*, *MRPS27*, *DET1*, *SH3PXD2A*, *NKX3-1*, *AGTR1*, *SOX6*, *INPP5F*, *JUN*, *LRRC59*, *DOCK1*, *CIAPIN1*, *HIVEP2*, *CEBPB*
hsa-let-7b-5p	*CPEB3*, *CYP2J2*, *TLR4*, *AKT2*, *EZH2*, *HRAS*, *LIN28B*, *CCND2*, *CDC34*, *RDH10*, *CDC25A*, *CCNA2*, *HMGA1*, *LIN28A*, *E2F2*, *CPEB1*, *ANAPC1*, *NRAS*, *IRS2*, *AGO1*, *CTHRC1*, *IGF2BP2*, *PRDM1*, *NR2E1*, *IFNB1*, *HMGA2*, *MTPN*, *CDK6*, *IGF1R*, *TNFRSF10B*, *IGF2BP1*, *COL3A1*, *CCND1*, *TGFBR1*, *LGR4*
hsa-let-7c-5p	*CEBPB*, *STAT3*, *COPS8*, *MYC*, *HSPA4*, *RICTOR*, *TRIM71*, *NRAS*, *ITGB3*, *AGO1*, *PBX2*, *HMGA2*, *IL10*, *IGF1R*, *CDC25A*, *GPS1*, *TRIB2*, *BCL2L1*, *TNFRSF10B*, *IL6*, *MAP4K3*, *CASP3*, *NUMB*, *TGFBR1*, *MTOR*, *COPS6*
hsa-miR-3127-5p	*PHLPP1*, *PHLPP2*, *INPP5J*, *INPP4A*
hsa-miR-423-5p	*FAM3A*, *TFF1*

## Data Availability

All data generated during this study are included in the article. Further inquiries should be addressed to Shuailin Li and Nóra Szentmáry.

## Supplementary Materials

The following supporting information can be downloaded at https://www.mdpi.com/article/10.3390/biology15151239/s1: Figure S1: Principal component analysis (PCA) of corneal and conjunctival impression cytology (IC) samples. The first principal component (PC1) and the second principal component (PC2) explained 40.1% and 18.4% of the total variance, respectively; Figure S2: Protein–protein interaction (PPI) network of differentially expressed genes (DEGs) upregulated in corneal impression cytology (IC) samples compared with conjunctival IC samples. The network was generated using the STRING database (https://string-db.org; accessed on 20 April 2026) and visu-alized with Cytoscape (version 3.10.4). Nodes represent proteins encoded by the identified DEGs, while edges indi-cate known or predicted protein–protein interactions; Figure S3: Protein–protein interaction (PPI) network of differentially expressed genes (DEGs) upregulated in conjunctival impression cytology (IC) samples compared with corneal IC samples. The network was generated using the STRING database (https://string-db.org; accessed on 20 April 2026) and visu-alized with Cytoscape (version 3.10.4). Nodes represent proteins encoded by the identified DEGs, while edges indi-cate known or predicted protein–protein interactions.

## Abbreviations

BP	Biological process
CC	Cellular component
cDNA	Complementary DNA
DEGs	Differentially expressed genes
FC	Fold change
FDR	False discovery rate
GO	Gene Ontology
GST	Glutathione S-transferase
GUSB	Glucuronidase beta
IC	Impression cytology
ISGs	Interferon-stimulated genes
KEGG	Kyoto Encyclopedia of Genes and Genomes
MCC	Maximal clique centrality
MF	Molecular function
miRNA	Micro RNA
mRNA	Messenger RNA
PCA	Principal component analysis
PPI	Protein–protein interaction
RNU6	RNA U6
RPMMM	Reads per million mapped miRNAs
RT-qPCR	Real-time quantitative PCR
TBP	TATA-box binding protein
VST	Variance-stabilizing transformation
