# Supplementary material for: mRNA and microRNA Expression Profile of Corneal and Conjunctival Impression Cytology Samples
Source: Biology (Basel). 2026 Jul 27;15(15):1239. doi: 10.3390/biology15151239 (PMC13463880; doi:10.3390/biology15151239)
Supplement: Supplementary file 1 [file biology-15-01239-s001.zip › biology-4421870-supplementary.pdf]

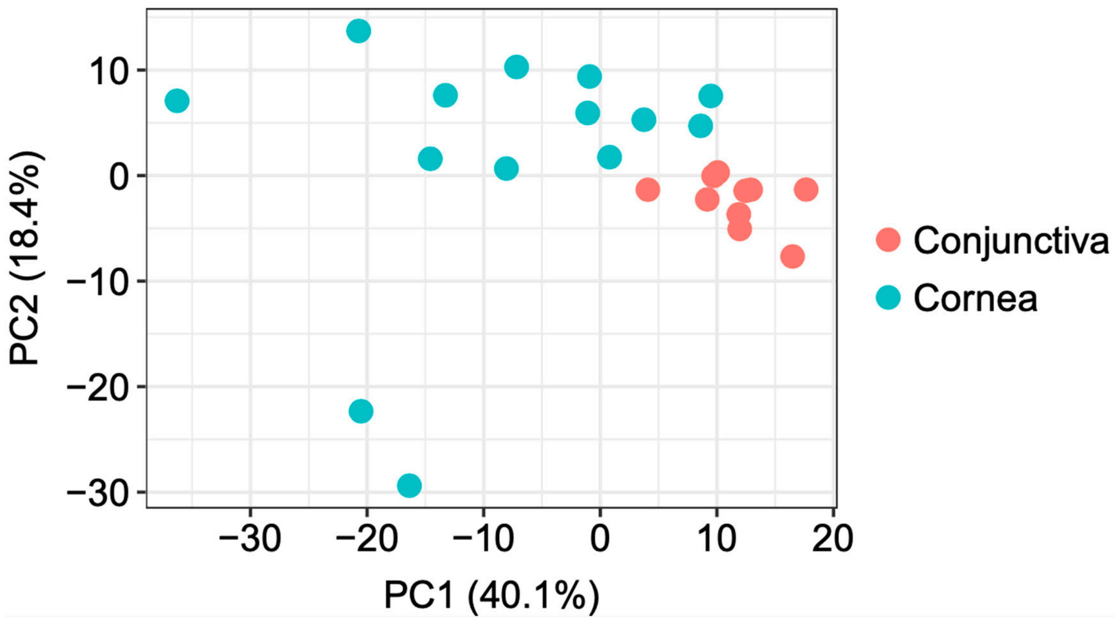

Figure S1. Principal component analysis (PCA) of corneal and conjunctival impression cytology (IC) samples. The first principal component (PC1) and the second principal component (PC2) explained 40.1% and 18.4% of the total variance, respectively.

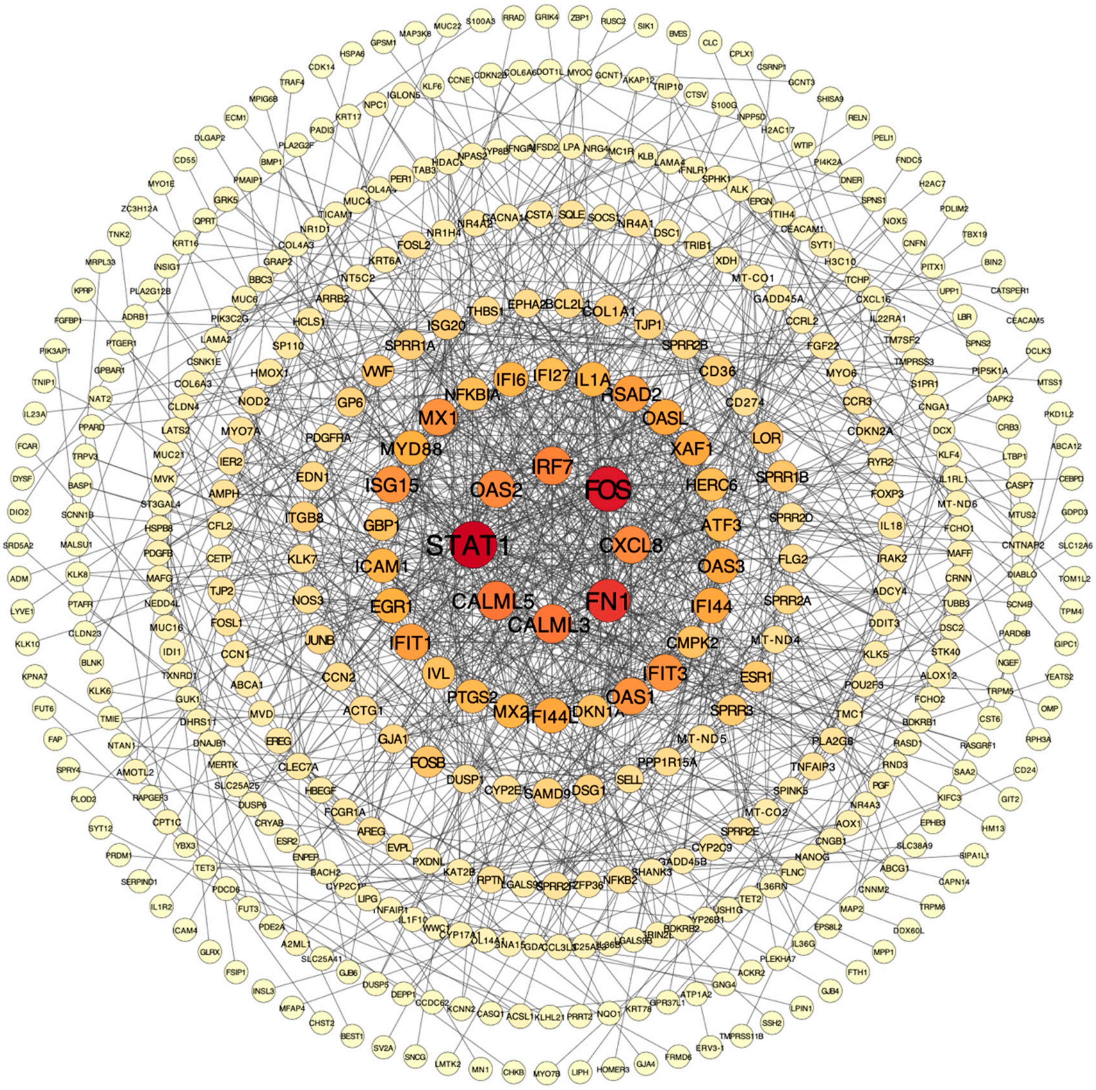

Figure S2. Protein–protein interaction (PPI) network of differentially expressed genes (DEGs) upregulated in corneal impression cytology (IC) samples compared with conjunctival IC samples. The network was generated using the STRING database (https://string-db.org; accessed on April 20, 2026) and visualized with Cytoscape (version 3.10.4). Nodes represent proteins encoded by the identified DEGs, while edges indicate known or predicted protein–protein interactions.

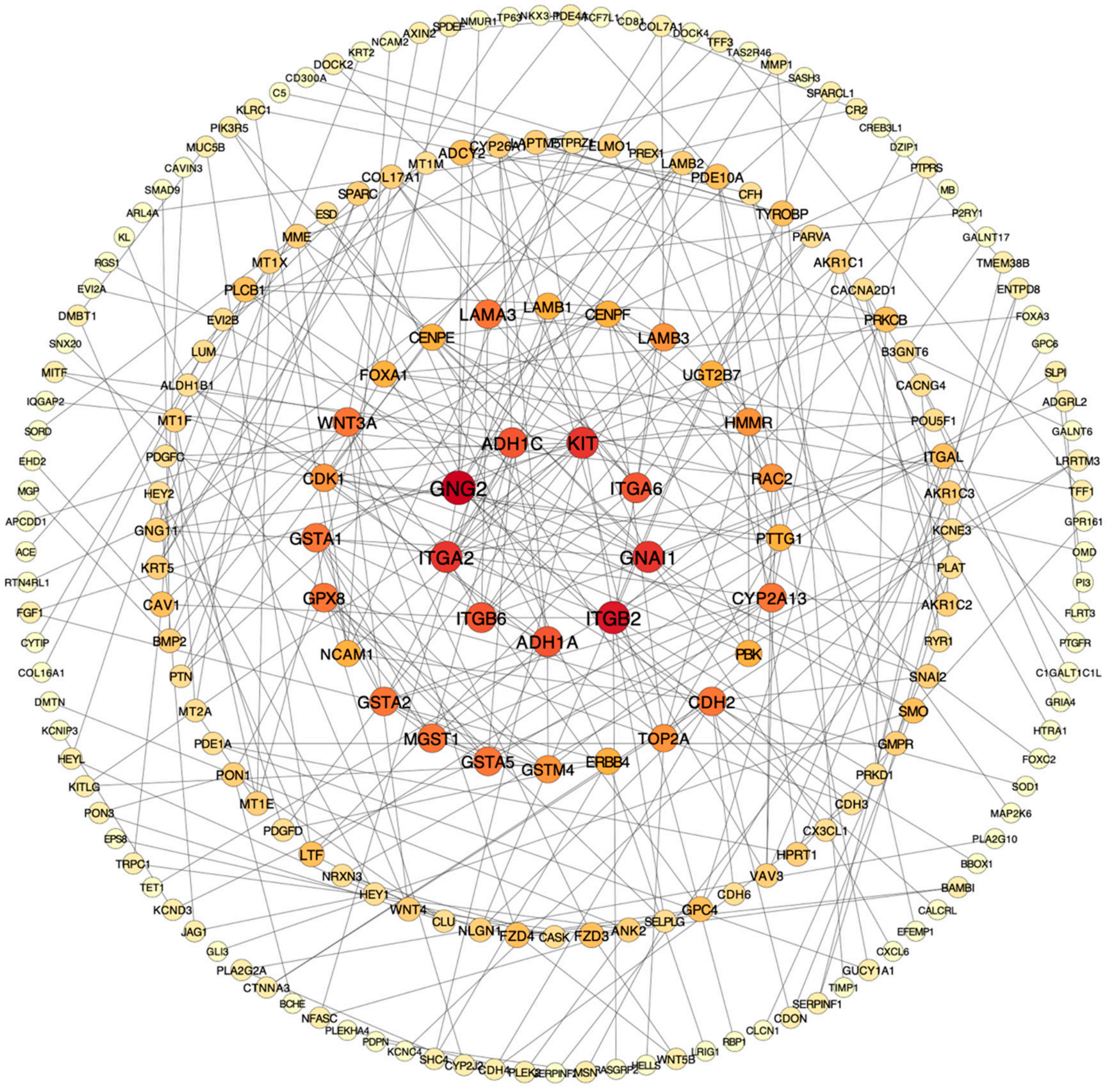

Figure S3. Protein–protein interaction (PPI) network of differentially expressed genes (DEGs) upregulated in conjunctival impression cytology (IC) samples compared with corneal IC samples. The network was generated using the STRING database (https://string-db.org; accessed on April 20, 2026) and visualized with Cytoscape (version 3.10.4). Nodes represent proteins encoded by the identified DEGs, while edges indicate known or predicted protein–protein interactions.
